# Effect of Continuous Feeding of *Ayu-Narezushi* on Lipid Metabolism in a Mouse Model of Metabolic Syndrome

**DOI:** 10.1155/2021/1583154

**Published:** 2021-09-06

**Authors:** Takeshi Nishida, Koichi Tsuneyama, Yasuhiko Tago, Koji Nomura, Makoto Fujimoto, Takahiko Nakajima, Akira Noguchi, Takashi Minamisaka, Hideki Hatta, Johji Imura

**Affiliations:** ^1^Department of Diagnostic Pathology, Graduate School of Medicine and Pharmaceutical Sciences, University of Toyama, 2630 Sugitani, Toyama 930–0194, Japan; ^2^Department of Pathology and Laboratory Medicine, Institute of Biomedical Sciences, Tokushima University Graduate School, 3–18–15 Kuramoto, Tokushima 770–8503, Japan; ^3^Fisheries Research Institute, Toyama Prefectural Agricultural, Forestry & Fisheries Research Center, 364 Takatsuka, Namerikawa 936–8536, Japan; ^4^Department of Japanese Oriental Medicine, Graduate School of Medicine and Pharmaceutical Sciences, University of Toyama, 2630 Sugitani, Toyama 930–0194, Japan

## Abstract

*Ayu-narezushi*, a traditional Japanese fermented food, comprises abundant levels of lactic acid bacteria (LAB) and free amino acids. This study aimed to examine the potential beneficial effects of *ayu-narezushi* and investigated whether *ayu-narezushi* led to improvements in the Tsumura Suzuki obese diabetes (TSOD) mice model of spontaneous metabolic syndrome because useful LAB are known as probiotics that regulate intestinal function. In the present study, the increased body weight of the TSOD mice was attenuated in those fed the *ayu-narezushi*-comprised chow (*ayu-narezushi* group) compared with those fed the normal rodent chow (control group). Serum triglyceride and cholesterol levels were significantly lower in the *Ayu-narezushi* group than in the control group at 24 weeks of age. Furthermore, hepatic mRNA levels of carnitine-palmitoyl transferase 1 and acyl-CoA oxidase, which related to fatty acid oxidation, were significantly increased in the *ayu-narezushi* group than in the control group at 24 weeks of age. In conclusion, these results suggested that continuous feeding with *ayu-narezushi* improved obesity and dyslipidemia in the TSOD mice and that the activation of fatty acid oxidation in the liver might contribute to these improvements.

## 1. Introduction

Metabolic syndrome (MS) is a serious global health problem that is expected to worsen in the future because of the increasing prevalence of obesity, sedentary lifestyles, and unbalanced diets [1–3]. MS initiates with visceral fat accumulation and progresses to insulin resistance, abnormal lipid metabolism, hypertension, and various other diseases throughout the body [4]. Furthermore, MS may lead to/related to type 2 diabetes mellitus, vascular inflammation, atherosclerosis, and renal, liver, and heart diseases. Although the pathogenesis of MS is very complicated and controversial, the imbalance of the gut microbiota, i.e., dysbiosis, is suggested to be associated with MS [5–7]. Numerous studies have reported the beneficial effects of probiotics in improving dysbiosis, and probiotics have been increasingly introduced as health-promoting supplements in our daily diet. In fact, beneficial commensal microbes, such as *Bifidobacterium* spp. and *Lactobacillus* spp. have long been consumed as supplements [8, 9].

Many traditional fermented foods and beverages worldwide, such as yoghurt and kimuchi, have been reported as probiotic sources [10, 11]. *Narezushi*, one of the traditional fermented foods that is also a prototype of sushi in modern Japanese cuisine, is mainly made from salted and fermented rice and various fish types, and *Funazushi* made from crucian carp in Shiga prefecture is the most famous *narezushi* in Japan. In Toyama prefecture, *ayu-narezushi* is known since olden times, and it is made from boiled rice, malted rice, and salted *ayu* (*Plecoglossus altivelis*). Nomura et al. [12] analyzed the ingredients of *ayu-narezushi* made by the method handed down and reported that it comprised high levels of lactic acid bacteria (LAB) and free amino acids (FAAs), as shown in Table S1 (Supplementary Materials, SM1). Certain LAB and *Bifidobacteria* are considered as probiotics based on their health-promoting effects involving metabolism and immunity [13]. LAB reportedly lowers serum cholesterol levels in animals and humans [14, 15]. Furthermore, certain FAAs have been recognized for their biological activities; e.g., taurine has effective actions on MS, which include reducing triglycerides to prevent obesity and improving insulin resistance to regulate glucose metabolism [16]. The abovementioned studies have, therefore, focused on the relationship between pathophysiology and specific components, such as LAB and FAA, regarding improving disorders. Accordingly, we considered that *ayu-narezushi* containing abundant LAB and FAAs might become not only a mere traditional food but also a novel candidate for functional food as probiotics.

Tsumura Suzuki obese diabetes (TSOD) mice models (TSOD mice) are polygenic models of spontaneous type 2 diabetes mellitus in which the mice develop moderate degrees of obesity, which is particularly apparent in animals older than 11 weeks of age [17, 18]. Male TSOD mice exhibit glycosuria, hyperglycemia, and hyperinsulinemia within the absence of any special treatment [19] and are valuable models for examining disease mechanisms and drug efficacy in MS treatment. Therefore, we hypothesized that feeding TSOD mice with *ayu-narezushi* is abundant in LAB and FAAs improves the pathophysiology of MS.

This study aimed to investigate the effect of *ayu-narezushi* on MS through the feeding experience of TSOD mice. We also examined a part of the action mechanism, improving lipid metabolism in mice by using gene expression analysis.

## 2. Materials and Methods

### 2.1. Preparation of Ayu-Narezushi

The main components of *ayu-narezushi* are ayu (*Plecoglossus altivelis*), cooked rice, and salt. Briefly, the internal organs and gills of the fish caught from the Shou River in Toyama prefecture were removed and soaked in natural salt at 5°C in a refrigerator for 3 weeks. Thereafter, the meat was rinsed under running water for 5 minutes to remove excess salt and slime. Then, cooked rice and malted rice were placed in the bottom of a keg and layered on top with the meat and cooked rice, in order; this procedure was repeated 7-8 times to create layers of rice and meat. The inner lid of the keg was then placed, and weights were placed on top of the lid. The keg was preserved in a cold and dark place for fermentation (temperature range: 5°C−10°C), and *ayu-narezushi* can be consumed after approximately 1 month. In the present study, *ayu-narezushi* that was fermented for 60 days in the keg was used.

### 2.2. Experimental Animals

Overall, 20 male TSOD mice at 4 weeks of age were purchased from the Institute for Animal Reproduction (Ibaraki, Japan). During the acclimation period, the animals were provided a normal rodent chow with Labo MR Stock (Nosan Corporation, Yokohama, Japan) and chlorinated water *ad libitum*. The mice were housed in plastic cages in a nonbarrier-sustained animal room maintained at 22°C ± 2°C with 60% ± 10% relative humidity and a 12/12 h light/dark cycle. After acclimation, the TSOD mice were weighted and assigned to two groups (*n* = 10): the control group that was fed the normal chow and *ayu-narezushi* group that was fed the chow comprising 10% (w/w) *ayu-narezushi*, which was prepared by mixing the normal chow with *ayu-narezushi* including the meat, cooked rice, and malted rice. The experimental diet was fed to the TSOD mice starting from 5 weeks of age. During the experiment, there was no notable difference in food intake between the two groups. The study was performed in accordance with the animal experiment guidelines specified by the University of Toyama. All experimental protocols were approved by the Committee on Animal Experimentation of the University of Toyama (A2015MED-54).

During the experiment, the body weights of all mice were measured once a week from the start of the experiment (5 weeks of age) to the end of the experiment (24 weeks of age). For histological and biochemical analyses, 5 mice in each group were sacrificed at 8 and 24 weeks of age. Additionally, after anesthesia with sodium pentobarbital, blood samples were collected from the posterior vena cava and centrifuged at 1,200 × g for 15 minutes to collect serum. The samples were maintained at −80°C until further use for biochemical analysis. Additionally, the main internal organs, including the liver, pancreas, kidney, lungs, and spleen, as well as visceral fat, were rapidly excised and rinsed in ice-cold saline, followed by fixation in 10% neutral-buffered formalin, and embedded in paraffin for histopathological analysis. Remaining liver and visceral fat tissues were also stored at −80°C until further use for gene expression analysis.

### 2.3. Histological and Biochemical Analyses

The formalin-fixed paraffin-embedded tissues were processed using standard procedures, and 4 *μ*m-thick serial sections were stained with hematoxylin and eosin for histological analysis using an optical microscope (Olympus BX41; Olympus Corporation, Tokyo, Japan). The number of visceral adipocytes was determined using the WinROOF image analysis software (Mitani Corporation, Fukui, Japan). The digital images of a minimum of three fields of hematoxylin-/eosin-stained slides of visceral fat were captured at 200x magnification using a digital camera (Olympus DP21, Olympus Corporation, Tokyo, Japan), and the software counted the number of visceral adipocytes.

The levels of serum triglycerides and cholesterol were measured using commercial assay kits (LabAssay™ Triglyceride: Code No. 290-63701 and LabAssay™ Cholesterol: Code No. 294-65801; Wako Pure Chemical Industries, Osaka, Japan) according to the manufacturer's protocol; these levels in the serum collected from both groups at 8 and 24 weeks of age were compared.

### 2.4. RNA Preparation and Real-Time PCR

The mRNA expression levels of three genes related to fatty acid oxidation (peroxisomal proliferator-activated receptor *α*; PPAR*α*, acyl-CoA oxidase; ACO, and carnitine-palmitoyl transferase; CPT1) were assessed by real-time PCR. Total RNA was isolated from the liver and visceral fat samples by using Isogen2 reagent (Nippon Gene, Japan). Complementary DNA was synthesized from 500 ng of total RNA by using a ReverTra Ace® qPCR RT Master Mix with a gDNA Remover (Toyobo, Japan). PCR reactions and analyses were carried out using a LightCycler® Nano System (Roche, Japan) with denaturation for 10 min at 95°C, followed by 45 PCR cycles of denaturation at 95°C for 10 s, annealing at 60°C for 10 s, and extension at 72°C for 15 s. All primers used for analysis were designed by Eurofins Genomics (Japan). The primer sequence details are presented in Table 1 [20]. The amount of mRNA was calculated using glyceraldehyde-3-phosphate dehydrogenase as an endogenous control.

### 2.5. Statistical Analysis

Values were expressed as means ± standard deviation. Means were compared using Student's *t*-test. A *P* value of <0.05 was considered statistically significant.

## 3. Results

### 3.1. Changes in Body Weight in the Ayu-Narezushi and the Control Groups from 4 to 24 Weeks of Age

**Figure 1:**
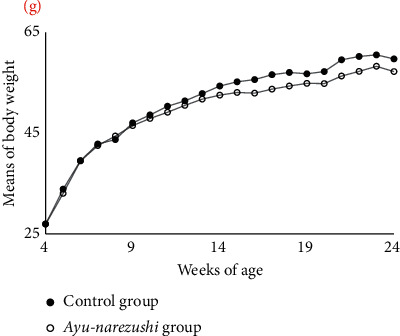
Comparison of changes in mean body weight between the *ayu-narezushi* and the control groups from 4 to 24 weeks of age. The mean body weight of the *ayu-narezushi* group (*n* = 5, white circles) was lower than that of the control group (*n* = 5, black circles) over 10 weeks of age. This trend continued until 24 weeks of age, with a gradual increase in the difference in mean body weight between the two groups over the weeks.

The mean body weight in both the *ayu-narezushi* and control groups increased gradually from 4 to 24 weeks of age, when the experiment was concluded. However, there was a tendency of attenuation in the increase of mean body weight starting approximately at 11 weeks of age in the *ayu-narezushi* group compared with the control group (Figure 1). In statistical analysis, there was not the statistical significant difference between *ayu-narezushi* and control group.

### 3.2. Comparison of Histopathological Findings in Visceral Fat in the Two Groups

**Figure 2:**
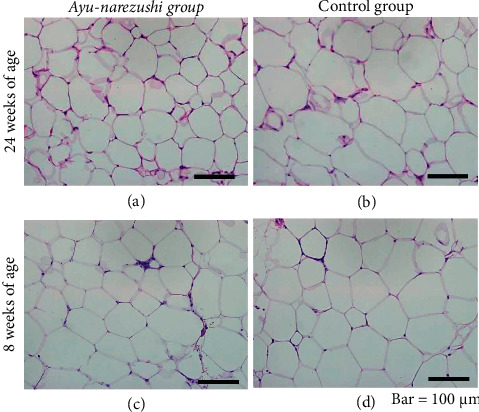
Micrographs of visceral fat in the Tsumura Suzuki obese diabetes (TSOD) mice that were fed the *ayu-narezushi* (a, c) or control (b, d) chow. The samples are from mice at 8 and 24 weeks of age. The size of the visceral adipocytes in the *ayu-narezushi* group (a) is remarkably smaller than that of the control group (b) at 24 weeks of age, although they are comparable between the two groups (c, d) at 8 weeks of age (×200 magnification). Bar = 100 *μ*m.

The size of visceral adipocytes was remarkably smaller in the *ayu-narezushi* group than in the control group at 24 weeks of age (Figures 2(a) and 2(b)); however, no remarkable difference was observed in their size between the two groups at 8 weeks of age (Figures 2(c) and 2(d)). The quantification of the images captured at identical magnification revealed that the mean number of visceral adipocytes at 24 weeks of age was significantly higher in the *ayu-narezushi* group (100.3 ± 18.2 cells) than in the control group (85.2 ± 14.5 cells) (Table 2, *P* < 0.05).

On the other hand, no remarkable differences were observed in other organs, including the liver and spleen, between the *ayu-narezushi* and control groups.

### 3.3. Serum Levels of Triglycerides and Cholesterol in the Ayu-Narezushi and the Control Groups

**Figure 3:**
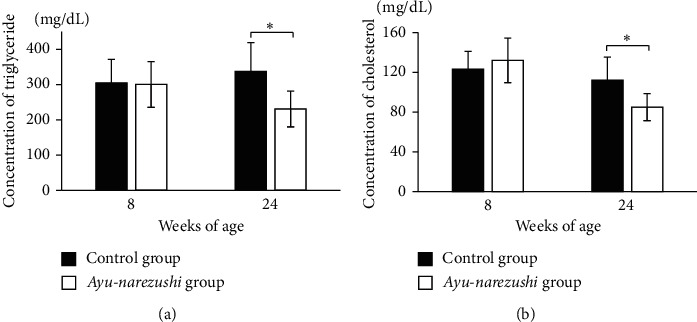
Comparison of serum concentrations of triglycerides (a) and cholesterol (b) at 8 and 24 weeks of age in TSOD mice fed the *ayu-narezushi*-containing or the control chow. Serum triglyceride levels are significantly lower at 24 weeks of age in the *ayu-narezushi* group (*n* = 5, white column) compared with the control group (*n* = 5, black column) (a). Serum cholesterol levels are significantly lower at 24 weeks of age in the *ayu-narezushi* group (*n* = 5, white column) than the control group (*n* = 5, black column) (b). The serum triglyceride and cholesterol levels are comparable between the two groups at 8 weeks of age. Variables are expressed as means ± standard deviation. Means were compared using Student's *t* test. ^*∗*^*P* < 0.05.

The serum triglyceride and cholesterol levels, which were not significantly different at 8 weeks of age between the two groups, were significantly lower in the *ayu-narezushi* group than in the control group at 24 weeks of age (Figures 3(a) and 3(b), *P* < 0.05).

### 3.4. Validation of Gene Expression with Real-Time PCR

**Figure 4:**
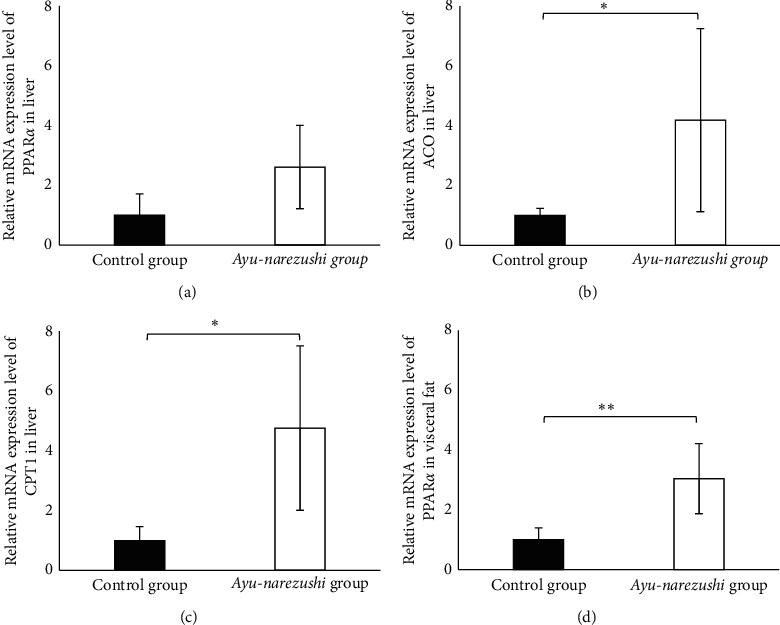
PPAR*α* (a), ACO (b), and CPT1 (c) mRNA levels in liver and PPAR*α* (d) mRNA levels in visceral fat. Each tissue was isolated at 24 weeks of age. The mRNA levels were determined by real-time PCR, normalized to glyceraldehyde-3-phosphate dehydrogenase. The data represent the mean ± standard deviation of 5 animals. ^*∗*^Significantly different from the control group with ^*∗*^*P* < 0.05; ^*∗∗*^*P* < 0.01. PPAR, peroxisome proliferator-activated receptor; ACO, acyl-CoA oxidase; and CPT1, carnitine-palmitoyl transferase.

Hepatic mRNA expression levels of PPAR*α* tended to increase in the *ayu-narezushi* group compared with the control group at 24 weeks of age (Figure 4(a)), although significant difference were not observed. In addition, hepatic mRNA expression levels of ACO and CPT1 were significantly increased in the *ayu-narezushi* group compared with the control group at 24 weeks of age (Figures 4(b) and 4(c), *P* < 0.05). These data suggest that an overexpression of ACO and CPT1 induced improvement of lipid metabolism through activation of fatty acid oxidation.

The visceral fat mRNA expression level of PPAR*α* was significantly increased in the *ayu-narezushi* group compared with the control group at 24 weeks of age (Figure 4(d), *P* < 0.01).

## 4. Discussion

In the current study, we investigated the effect of *ayu-narezushi* on conditions associated with MS including weight gain and hyperlipidemia using TSOD mice feeding *ayu-narezushi* because *ayu-narezushi* comprised abundant levels of LAB and FAA that are extensively known for their probiotic activity. During the experiment period, we found that the increase in mean body weight slowed down gradually from 11 to 24 weeks of age in mice that were fed the *ayu-narezushi*-comprising chow compared with that in mice fed the control chow. The size of the visceral adipocytes was smaller in the *ayu-narezushi* group than in the control group at 24 weeks of age. Furthermore, the serum triglyceride and cholesterol levels were significantly lower in the *ayu-narezushi* group. Overall, these data suggested that the long-term consumption of *ayu-narezushi* is beneficial for TSOD mice.

Next, we investigated the action mechanism that lipid metabolism was improved in the *ayu-narezushi* group using gene expression analysis. In this study, we showed that the hepatic mRNA expression level of PPAR*α* tended to increase in the *ayu-narezushi* group, although significant difference was not observed. Furthermore, hepatic mRNA levels of PPAR*α* target genes, such as CPT1 and ACO, were significantly increased in the same group. PPAR*α* is expressed principally in the liver and is a key regulator of lipid metabolism [21, 22]. PPAR*α* regulates the expression of many genes, such as ACO and CPT1, that were involved in fatty acid oxidation [23, 24]. Accordingly, these data suggested that *ayu-narezushi* induced fatty acid oxidation in the liver of TSOD mice.

In addition, we also found that visceral fat mRNA expression levels of PPAR*α* were significantly increased in the *ayu-narezushi* group at 24 weeks of age. Although PPAR*α* is expressed at high levels in the liver, Takahashi et al. [25] reported that overexpression of PPAR*α* in obese mice adipose tissue improves insulin sensitivity and suggested that PPAR*α* activation in adipose tissue contributes to the improvement of glucose metabolism disorders via the enhancement of free fatty acid metabolism. Although further studies are needed to investigate, PPAR*α* activation in visceral fat of TSOD mice feeding *ayu-narezushi* might play an important role in the improvement of disease related to lipid metabolism dysfunction.

In this study, the specific component candidate substances of *ayu-narezushi* that were related to these beneficial effects remain unclear. Nomura et al. [12] have reported that *ayu-narezushi* fermented for 60 days comprised LAB (3.7 × 10^7^ cfu/g), organic acids (mainly lactic acid; 3,114 mg/100 g), and FAA (1,169 mg/100 g). In the further study, they had also found that the LAB sufficiently fermented *ayu-narezushi* over 40 days primarily comprised *L. sakei* (90%). Therefore, the *ayu-narezushi* in the current study, which was fermented longer (60 days), should comprise comparable levels of *L. sakei*. Although few studies have investigated this bacterium, Lim et al. [26] have reported that *L. sakei* OK67 ameliorated high-fat-diet-induced hyperglycemia and obesity by reducing inflammation and increasing the expression of colon tight junction proteins in mice. This LAB species might be functionally effective as an antiobesity agent in TSOD mice.

Recently, studies have revealed the novel functional roles of D-amino acids. Amino acids have an alpha-carbon that is connected to four functional groups, including amine and carboxyl groups, a hydrogen, and a side chain. Amino acids, except for glycine, have two stereoisomers: levorotatory (L) and dextrorotatory (D). Although L-amino acids are essential for life as the building blocks of proteins, D-amino acids reportedly impact important physiological events in mammals, such as neurotransmission, testosterone synthesis, and fertilization [27–29]. We have performed mass spectrometry analysis to quantify the L- and D-amino acids [30] and found six D-amino acids such as D-Ala and D-Asp in *ayu-narezushi* (Figure S1: Supplementary Materials, SM2). Kato and Oikawa [31] have also reported that *L. sakei* LK-145 produces high levels of D-amino acids. Therefore, it is possible that the D-amino acids in *ayu-narezushi* contribute to the improvements in the body weight and serum levels of triglycerides and cholesterol in TSOD mice. However, further investigation is warranted to identify the specific D-amino acids in *ayu-narezushi* and their functional roles.

Two studies have examined the components of the gut microbiome in TSOD mice at different ages. One study comparing the intestinal flora of TSOD mice with that of control mice at 5 and 12 weeks of age has observed that some species were detected only in the TSOD mice such as the genera *Turicibacter* and SMB53, a member of the Clostridiaceae family, and suggested that these bacteria contribute to type 2 diabetes in TSOD mice [32]. The second study has reported that there was a correlation among gut dysbiosis, short-chain fatty acids, and MS in 24-week-old TSOD mice [33]. Enriched levels of Gram-positive bacteria and corresponding decreases in Gram-negative bacteria were also detected in TSOD mice. Moreover, the abundance of *Bacteroidetes* spp. was decreased, whereas that of the *Firmicutes* spp. was increased, and the total plasma short-chain fatty acid level was significantly lower in the TSOD mice than that of the controls. These results suggested the dysbiosis of the gut microbiome in TSOD mice as early as 12 weeks of age, which was observed until 24 weeks of age. LAB intake has been reported to modulate the gut microbiota in some studies [34, 35]. Although *L. sakei* in *ayu-narezushi* might have improved the gut microbiome of TSOD mice, further studies clarifying the association between *ayu-narezushi* feeding and dysbiosis in this animal model are warranted.

## 5. Conclusions

Feeding TSOD mice with *ayu-narezushi*, which comprises abundant levels of LAB and FAA, improved the change in body weight and serum levels of triglycerides and cholesterol. In these mice, it was considered that the activation of fatty acid oxidation in the liver had contributed to the improvement of diseases related to MS. Traditional fermented food items comprising LAB, such as yoghurt and sauerkraut, are available worldwide. The continuous use of drugs for the prevention of MS is not ideal. Therefore, we propose that the continuous feeding of traditional fermented foods like *ayu-narezushi* may be expected to have great potential for the improvement of MS.

## Figures and Tables

**Table 1:** Primer pair sequences used for real-time PCR.

Target	Sequence	
	Forward	Reverse
PPAR*α*	ATTTGCTGTGGAGATCGGCC	TGGTTGCTCTGCAGGTGGAG
ACO	TCTTCTTGAGACAGGGCCCAG	GTTCCGACTAGCCAGGCATG
CPT1	CTTCCAAGGCAGAAGAGTGGG	GAACCTTGGCTGCGGTAAGAC
GAPDH	ATGACATCAAGAAGGTGGTG	CATACCAGGAAATGAGCTTG

PPAR: peroxisome proliferator-activated receptor, ACO: acyl-CoA oxidase. CPT: carnitine-palmitoyl transferase, GAPDH: glyceraldehydes-3-phosphate dehydrogenase.

**Table 2:** Comparison of the mean number of visceral adipocytes between the *ayu-narezushi* and the control groups at 8 and 24 weeks of age.

	8 weeks	24 weeks
Control group	59.2 ± 12.6	85.2 ± 14.5
*Ayu-narezushi* group	63.9 ± 5.3	100.3 ± 18.2^*∗*^

Data are expressed as mean ± standard deviation. Means were compared using Student's *t*-test. ^*∗*^*P* < 0.05 vs. control group at 24 weeks of age.

## Data Availability

The data used to support the findings of this study are available from the corresponding author upon request.

## Supplementary Materials

Supplementary Materials Additional file. PDF: SM1. Table S1 shows microbial count, organic acids, and free amino acids of *ayu-narezushi* ripened. The data have been excerpted from the work of Nomura et al. [12]. SM2. Figure S1 shows the chromatograms of 6 kinds of D-amino acids detected in *ayu-narezushi*.
