# Supplementary material for: Effect of Continuous Feeding of Ayu-Narezushi on Lipid Metabolism in a Mouse Model of Metabolic Syndrome
Source: ScientificWorldJournal. 2021 Sep 6;2021:1583154. doi: 10.1155/2021/1583154 (PMC8440109; doi:10.1155/2021/1583154)
Supplement: Supplementary Materials — Additional file. PDF: SM1. Table S1 shows microbial count, organic acids, and free amino acids of ayu-narezushi ripened. The data have been excerpted from the work of Nomura et al. [12]. SM2. Figure S1 shows the chromatograms of 6 kinds of D-amino acids detected in ayu-narezushi. [file 1583154.f1.pdf]

Table S1: Microbial count, Organic acids, and Free amino acids of *ayu-narezushi* ripened. The data have been excerpted from Nomura et al. (*12*) Amino acids had not been distinguished between L- and D-Amino acids in the manuscript.

		Ripening periods (Days)						
		0 (Brined fish)	8	15	22	30	40	60
Microbial counts (cfu/g)	Lactic acid bacteria	1.9×10^5	9.8×10^7	3.2×10^8	4.3×10^8	3.2×10^8	1.8×10^8	3.7×10^7
Organic acid (mg/100g)	Lactic acid	195	1435	2206	2443	2409	2826	3114
	Acetic acid	0	84	116	163	190	235	192
	Succinic acid	0	30	39	41	35	44	47
	Pyruvic acid	0	180	202	236	217	34	41
	Formic acid	0	69	63	59	45	46	53
Amino acid (mg/100g)	Taurine	112	27	33	38	33	35	32
	Aspartic acid	15	13	34	12	50	68	83
	Threonine	11	15	31	45	39	47	54
	Serine	19	7	16	28	29	36	48
	Asparagine	N.D.	7	14	22	20	27	31
	Glutamic acid	22	41	99	131	118	154	173
	Glutamine	5	4	8	15	14	20	24
	Glycine	28	16	31	38	36	46	52
	Alanine	27	25	60	76	60	82	84
	Valine	13	17	39	49	42	53	57
	Methionine	8	18	33	49	43	49	53
	Proline	10	6	12	17	15	17	13
	Arginine	7	8	8	19	8	3	4
	Lysine	28	32	61	81	77	97	102
	Histidine	10	7	11	13	13	18	21
	Isoleucine	8	19	40	57	51	60	62
	Leucine	16	46	86	123	108	127	137
	Tyrosine	18	10	20	38	32	43	44
	Phenylalanine	10	22	40	56	50	60	72
	GABA	0	1	2	3	4	4	3
	Tryptophan	N.D.	N.D.	N.D.	11	11	13	N.D.

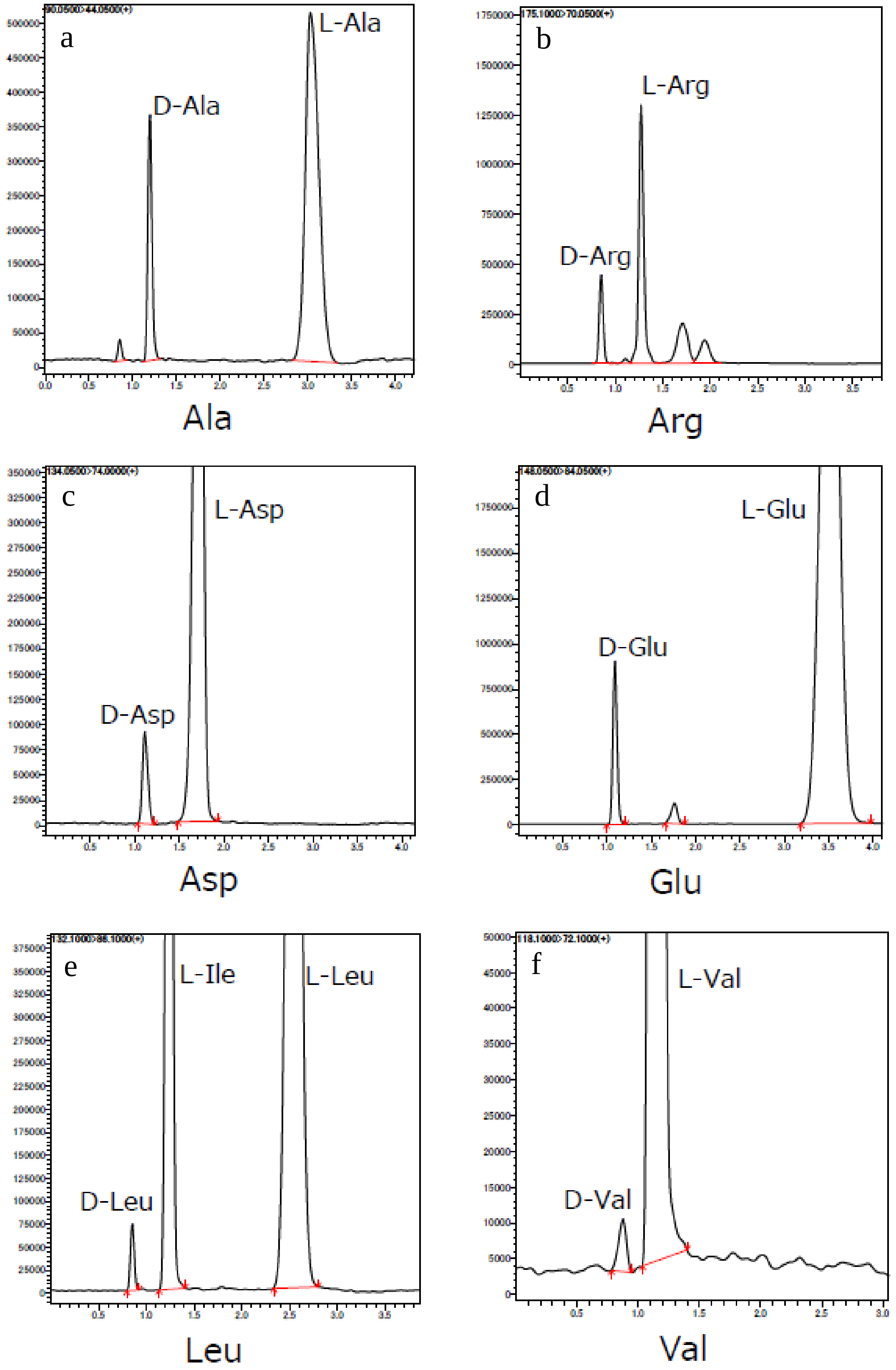

Figure S1: The chromatograms of 6 kinds of D-amino acids detected in *ayu-narezushi*. a: Alanine, b: Arginine, c: Aspartic Acid, d: Glutamic Acid, e: Leucine, f: Valine.
